# Response prediction of hepatocellular carcinoma undergoing transcatheter arterial chemoembolization: unlocking the potential of CT texture analysis through nested decision tree models

**DOI:** 10.1007/s00330-020-07511-3

**Published:** 2020-12-03

**Authors:** Jan Vosshenrich, Christoph J. Zech, Tobias Heye, Tuyana Boldanova, Geoffrey Fucile, Stefan Wieland, Markus H. Heim, Daniel T. Boll

**Affiliations:** 1 Department of Radiology, University Hospital Basel, Petersgraben 4, 4031 Basel, Switzerland; 2 Clarunis - University Center for Gastrointestinal and Liver Diseases, Petersgraben 4, 4031 Basel, Switzerland; 3 Department of Biomedicine, University Hospital Basel, University of Basel, Hebelstrasse 20, 4031 Basel, Switzerland; 4 sciCORE - Center for Scientific Computing, University of Basel, Klingelbergstrasse 50/70, 4031 Basel, Switzerland

**Keywords:** Hepatocellular carcinoma, Treatment outcome, X-ray computed tomography, Therapeutic chemoembolization, Decision trees

## Abstract

**Objectives:**

To investigate if nested multiparametric decision tree models based on tumor size and CT texture parameters from pre-therapeutic imaging can accurately predict hepatocellular carcinoma (HCC) lesion response to transcatheter arterial chemoembolization (TACE).

**Materials and methods:**

This retrospective study (January 2011–September 2017) included consecutive pre- and post-therapeutic dynamic CT scans of 37 patients with 92 biopsy-proven HCC lesions treated with drug-eluting bead TACE. Following manual segmentation of lesions according to modified Response Evaluation Criteria in Solid Tumors criteria on baseline arterial phase CT images, tumor size and quantitative texture parameters were extracted. HCCs were grouped into lesions undergoing primary TACE (VT-lesions) or repeated TACE (RT-lesions). Distinct multiparametric decision tree models to predict complete response (CR) and progressive disease (PD) for the two groups were generated. AUC and model accuracy were assessed.

**Results:**

Thirty-eight of 72 VT-lesions (52.8%) and 8 of 20 RT-lesions (40%) achieved CR. Sixteen VT-lesions (22.2%) and 8 RT-lesions (40%) showed PD on follow-up imaging despite TACE treatment. Mean of positive pixels (MPP) was significantly higher in VT-lesions compared to RT-lesions (180.5 vs 92.8, *p* = 0.001). The highest AUC in ROC curve analysis and accuracy was observed for the prediction of CR in VT-lesions (AUC 0.96, positive predictive value 96.9%, accuracy 88.9%). Prediction of PD in VT-lesions (AUC 0.88, accuracy 80.6%), CR in RT-lesions (AUC 0.83, accuracy 75.0%), and PD in RT-lesions (AUC 0.86, accuracy 80.0%) was slightly inferior.

**Conclusions:**

Nested multiparametric decision tree models based on tumor heterogeneity and size can predict HCC lesion response to TACE treatment with high accuracy. They may be used as an additional criterion in the multidisciplinary treatment decision-making process.

**Key Points:**

*• HCC lesion response to TACE treatment can be predicted with high accuracy based on baseline tumor heterogeneity and size.*

*• Complete response of HCC lesions undergoing primary TACE was correctly predicted with 88.9% accuracy and a positive predictive value of 96.9%.*

*• Progressive disease was correctly predicted with 80.6% accuracy for lesions undergoing primary TACE and 80.0% accuracy for lesions undergoing repeated TACE.*

**Supplementary Information:**

The online version contains supplementary material available at 10.1007/s00330-020-07511-3.

## Introduction

Hepatocellular carcinoma (HCC) which accounts for more than 90% of primary liver cancers is the sixth most common cancer regarding incidence and the fourth most common cause of cancer-related mortality worldwide [1–3]. In accordance with clinical practice guidelines, patients diagnosed with intermediate or advanced stage of neoplastic disease are not amenable to curative surgical resection but are allocated to loco-regional interventional treatment or, alternatively, protein kinase inhibitor therapy such as sorafenib [3, 4]. Particularly in Barcelona Clinic Liver Cancer (BCLC) stage B patients, transcatheter arterial chemoembolization (TACE) represents the standard of care in many institutions [3, 5, 6]. Furthermore, TACE is also the most widely used bridging therapy in BCLC stage A patients awaiting liver transplantation. The efficacy of TACE has been demonstrated in randomized control trials [7, 8]. Noticeable differences in overall survival however suggest that not all treated lesions will effectively respond to TACE, as HCC patients show a wide spectrum of potential short- and long-term outcomes following treatment [9].

In recent years, efforts have been made identifying biomarkers of HCCs potentially predicting lesion response to TACE treatment, aiming to facilitate a decision process whether a patient should undergo primary or repeated TACE or be treated by different means. Utilized predictive algorithms were based on either laboratory results and clinical scores [10] or imaging parameters derived from CT or MRI texture analysis [11, 12]. The advent of such concepts demonstrated the emerging trend towards precision medicine in patients with focal liver disease and depicted the general ability to extend the assessment of HCC lesions beyond current classification systems.

While already suggested prediction approaches take several parameters into account, they are generally only used individually and dichotomously. By nesting multiple factors into a decision tree model, potentially using varying thresholds of redundant factors at different locations within the decision tree, a further increase in accuracy of treatment response prediction may result.

The aim of our study was to investigate the value of histogram-based CT texture analysis–derived nested decision tree models for the prediction of HCC lesion response to TACE treatment according to modified Response Evaluation Criteria in Solid Tumors (mRECIST) criteria in order to demonstrate that accurate prediction of complete response and progressive disease prior to both primary and repeated TACE is feasible.

## Materials and methods

### Study sample

**Fig. 1:**
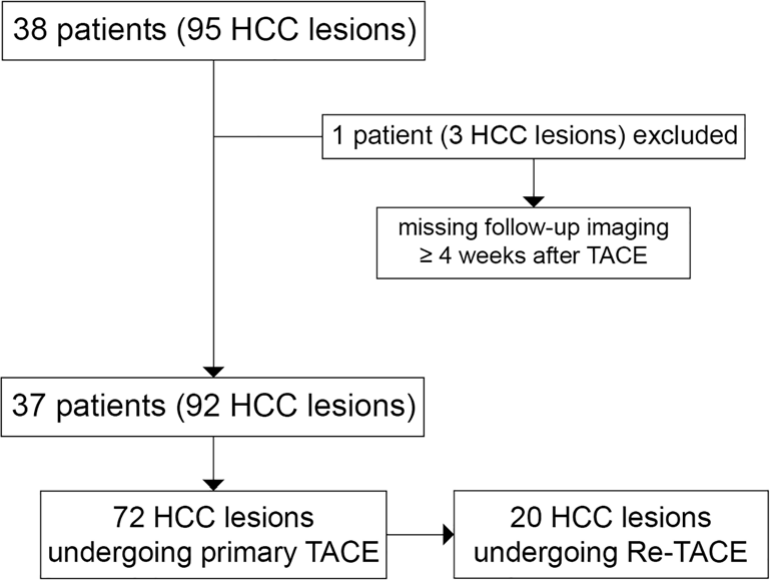
Flowchart of the study sample including exclusion criteria and grouping of lesions

This retrospective study was approved by the institutional review board; patients gave written informed consent. All patients treated with TACE and histopathologically proven HCC during the observation window between January 2011 and September 2017 were included. Exclusion criteria were (1) patients without baseline dynamic contrast-enhanced CT of the liver, (2) lack of follow-up CT imaging at the earliest 4 weeks after treatment, and (3) patients with non-diagnostic CT images. The final study sample consisted of 37 patients with a total of 92 individually treated HCC lesions (Fig. 1).

Both BCLC stage B patients undergoing palliative TACE and BCLC stage A patients being poor candidates for surgery or receiving TACE as a bridging treatment while awaiting liver transplantation were included. Due to the initial heterogeneity of disease stages, this study solely focused on target response prediction according to mRECIST and did not assess long-term outcome parameters, e.g., overall survival.

### CT imaging

All patients underwent four-phase CT according to the institutional standard liver imaging protocol. Imaging acquisitions were as follows: unenhanced, late arterial phase (AP), portal venous phase (PVP) and 3-min delayed phase (DP). CT examinations were acquired on a 128-slice (Somatom Definition Edge, Siemens Healthineers; tube settings 100 kV, 110 eff. mAs; collimation 128 mm × 0.6 mm, pitch 0.6, rotation time 0.5 s, slice thickness 1.5 mm) or 256-slice (Somatom Definition Flash, Siemens Healthineers; tube settings 100 kV, 65 eff. mAs; collimation 128 mm × 0.6 mm, pitch 0.6, rotation time 0.5 s, slice thickness 1.5 mm) scanner system. Following the unenhanced scan, 1.2 ml/kg of 370 mg I/ml iopromide (Ultravist® 370, Bayer Pharma) was injected intravenously at a flow rate of 4 ml/s by a power injector (Ulrich Medical). Using bolus tracking technique, AP images were acquired 18 s after reaching 100 HU in the descending aorta at the level of the celiac trunk. PVP and DP images were obtained 70 s and 180 s after reaching the scan initiation threshold. Timepoints of CT examinations were as follows: baseline imaging within 1 week prior to TACE, first follow-up imaging at 4 weeks after TACE and subsequent follow-ups at 3-month intervals.

### Transcatheter arterial chemoembolization procedure

TACE procedures were performed by one of two interventional radiologists with > 10 years of interventional experience (C.J.Z.). The right femoral artery was punctured using Seldinger technique, and a 4-French cobra or sidewinder catheter was inserted into the celiac trunk and common hepatic artery, respectively. In case of variant vascular anatomy, the superior mesenteric artery was catheterized additionally. To visualize feeding arteries of the tumor, digital subtraction angiography was performed and feeding vessels were superselectively intubated with a highly flexible 2.7-French microcatheter (ProGreat, Terumo). For embolization, doxorubicin-coated 100-μm beads (Tandem Beads, Embozene, now Boston Scientific) were slowly injected under fluoroscopic guidance up to a maximum dose of 150 mg doxorubicin. If stasis in the feeding vessel and disappearance of tumor staining was observed earlier, injection was terminated at a lower total doxorubicin dose. A closing DynaCT was obtained to assess treatment success.

### Image and texture analysis

Image datasets of all patients and timepoints were imported into mint Lesion™ 3.0 software (Mint Medical GmbH; commercially available) for post-processing. Every lesion was manually segmented on arterial phase images (axial plane, slice thickness 1.5 mm) according to mRECIST criteria by one radiologist with 2 years of experience in abdominal imaging (J.V.). All segmentations were reviewed by one radiologist specialized in abdominal imaging with > 15 years of experience (D.T.B.) and one radiologist specialized in abdominal imaging and interventional radiology with > 15 years of experience (C.J.Z.). Measurement discrepancies were resolved by consensus. CT texture analysis of segmented regions of interest (ROIs) was performed automatically by mint Lesion™ software based on gray-level histograms and included the following parameters: entropy, kurtosis, skewness, mean of positive pixels (MPP), and uniformity of positive pixel (UPP) distribution.

Response to TACE treatment as per mRECIST criteria was calculated after manual segmentation of lesions’ enhancing portions on baseline and follow-up CT examinations; mRECIST timepoint response evaluation criteria were the following: complete response (CR), disappearance of any intratumoral arterial enhancement; partial response (PR), at least 30% decrease in the sum of diameters of viable tumor; stable disease (SD), any cases not qualifying for either partial response or progressive disease; and progressive disease (PD), an increase of at least 20% in the sum of the diameters of viable tumor. If a lesion did not achieve CR after TACE and the institutional multidisciplinary gastrointestinal tumor board decided for repeated TACE treatment, a new baseline was set for the remaining enhancing portions of the lesion. This new baseline was used for subsequent response assessment after repeated TACE.

All CT texture analysis parameters, mRECIST timepoint responses, and lesion measurements specifically short- and long-axis diameters (in mm) and area (in mm^2^) were extracted.

### Statistical analysis and graphical visualization

Data was analyzed using SPSS 14 (IBM Corporation) for descriptive statistics and JMP® 14.0 (SAS Institute, Inc.) for calculation of prediction models, both commercially available.

HCCs were divided into two groups: (1) previously untreated lesions undergoing primary TACE (VT-lesions) and (2) lesions receiving repeated TACE (RT-lesions) due to incomplete response or progressive disease after the first TACE. This step was performed in order to differentiate if the first treatment sequence has any effect on the texture of the remaining viable tumor portions, and thus, a change in parameter thresholds needed for accurate response prediction. Baseline characteristics were compared using an independent Student’s *t* test, chi-square test, and Fisher’s exact test (significance level *p* < 0.05).

The two datasets were imported in the Prediction Profiler module in JMP®. CR or PD was defined as outcome parameters. The module created varying testing and confirmation datasets during the modeling process and calculated prediction models based on tumor size (area), surrounding hepatic parenchyma (cirrhotic vs non-cirrhotic liver), and parameters from CT texture analysis, resulting in four different decision tree models with automatically generated optimal discrimination thresholds for the respective parameters in each model: (1) VT-lesions with CR as goal of prediction, (2) VT-lesions with PD as goal of prediction, (3) RT-lesions with CR as goal of prediction, and (4) RT-lesions with PD as goal of prediction. Categorization of lesions was based on binary splitting. The minimum split size at each node was set at ten lesions to avoid overfitting.

Parameters’ contribution to the model, receiver operating characteristic (ROC) curve, and confusion matrices to depict model performance were generated. Based on confusion tables, positive prediction value (PPV), negative prediction value (NPV), and accuracies were calculated.

## Results

### Baseline characteristics

Of the 92 included lesions, 17 were found in women and 75 in men. Mean age at baseline imaging was 70.3 years (± 9.3, range 49–88). Eighty-seven percent (80/92) of lesions arose from cirrhotic livers, while 13% (12/92) were located in non-cirrhotic liver parenchyma. The mean number of HCC lesions per patient was 3 (range 1–7). Distribution of lesions between the different anatomical liver segments was heterogenous, and the majority was found in the right liver lobe (67/92, 67.8%), especially in segment VIII (31/92, 33.7%). All baseline characteristics are summarized in Table 1. Seventy-two HCC lesions in our study were treated with a single TACE, while 20 tumors required repeated treatment sessions (range 2–4 TACE treatments).

**Table 1:** Baseline characteristics of VT-lesions and RT-lesions showing complete or non-complete response to TACE

	VT-lesions			RT-lesions			All lesions (total)
	CR	Non-CR	Total	CR	Non-CR	Total	
No. of HCC lesions	38	34	72	8	12	20	92
Age (range)	69.7 (61–84)	73.9 (49–87)	71.7 (49–87)	64.0 (51–80)	66.1 (49–88)	65.3 (49–88)	70.3 (49–88)
Gender (%)							
Women	10 (26.3)	5 (14.7)	15 (20.8)	1 (12.5)	1 (8.3)	2 (10)	17 (18.5)
Men	28 (73.7)	29 (85.3)	57 (79.2)	7 (87.5)	11 (91.7)	18 (90)	75 (81.5)
Cirrhosis (%)							
Yes	37 (97.4)	23 (67.6)	60 (83.3)	8 (100)	12 (100)	20 (100)	80 (87)
No	1 (2.6)	11 (32.4)	12 (16.7)	0 (0)	0 (0)	0 (0)	12 (13)
BCLC stage (%)							
A	22 (57.9)	16 (47.1)	38 (52.8)	2 (25)	8 (66.7)	10 (50)	48 (52.2)
B	16 (42.1)	18 (52.9)	34 (47.2)	6 (75)	4 (33.3)	10 (50)	44 (47.8)
No. of HCC lesions per patient							
I	3.1 (1–7)	2.3 (1–7)	2.7 (1–7)	2 (1–4)	1.5 (1–2)	1.7 (1–4)	2.5 (1–7)
II	0 (0)	1 (2.9)	1 (1.4)	0 (0)	0 (0)	0 (0)	1 (1.1)
III	5 (13.2)	5 (14.7)	10 (13.9)	0 (0)	0 (0)	0 (0)	10 (10.9)
IVa	1 (2.6)	1 (2.9)	2 (2.8)	0 (0)	0 (0)	0 (0)	2 (2.2)
IVb	2 (5.3)	3 (8.8)	5 (6.9)	1 (12.5)	2 (16.7)	3 (15)	8 (8.7)
Liver segment (%)							
Left lobe	1 (2.6)	3 (8.8)	4 (5.6)	0 (0)	0 (0)	0 (0)	4 (4.3)
V	9 (23.7)	13 (38.2)	22 (30.6)	1 (12.5)	2 (16.7)	3 (15)	25 (27.2)
VI	9 (23.7)	2 (5.9)	11 (15.3)	1 (12.5)	2 (16.7)	3 (15)	14 (15.2)
VII	3 (7.9)	5 (14.7)	8 (11.1)	0 (0)	0 (0)	0 (0)	8 (8.7)
VIII	9 (23.7)	4 (11.8)	13 (18.1)	0 (0)	1 (8.3)	1 (5)	14 (15.2)
Right lobe	8 (21.1)	10 (29.4)	18 (25.0)	6 (75)	7 (58.3)	13 (65)	31 (33.7)
Area in mm^2^ (SD)	29 (76.3)	21 (61.8)	50 (69.4)	7 (87.5)	10 (83.3)	17 (85)	67 (67.8)
Long axis in mm (SD)	279.4 (± 385.2)	1938.3 (± 3131.2)	1062.8 (± 2308.6)	659.4 (± 864.3)	876.9 (± 1362.8)	789.9 (± 1167.2)	1003.4 (± 2110.8)
MPP (SD)	20.3 (± 11.2)	49.2 (± 34.1)	33.9 (± 28.6)	30.4 (± 19.5)	41.2 (± 31.0)	36.9 (± 26.9)	34.6 (± 28.1)
Entropy (SD)	248.2 (± 277.8)	104.8 (± 21.9)	180.5 (± 213.6)	95.3 (± 26.5)	91.1 (± 18.8)	92.8 (± 21.6)	161.4 (± 192.4)
Kurtosis (SD)	5.921 (± 0.61)	6.265 (± 0.41)	6.084 (± 0.55)	5.724 (± 0.94)	6.076 (± 0.68)	5.934 (± 0.79)	6.051 (± 0.61)
Skewness (SD)	3.0 (± 1.2)	3.6 (± 0.9)	3.3 (± 1.1)	3.2 (± 0.7)	3.5 (± 1.1)	3.4 (± 1.0)	3.3 (± 1.1)
Uniformity (SD)	0.0 (± 0.36)	− 0.1 (± 0.35)	− 0.1 (± 0.35)	0.1 (± 0.34)	− 0.2 (± 0.40)	− 0.1 (± 0.4)	0.1 (± 0.36)
Area in mm^2^ (SD)	0.0206 (± 0.009)	0.0160 (± 0.005)	0.0184 (± 0.008)	0.0268 (± 0.022)	0.0191 (± 0.009)	0.0222 (± 0.015)	0.0192 (± 0.010)

*VT-lesions* lesions undergoing primary TACE, *RT-lesions* lesions undergoing repeated TACE, *CR* complete response, *Non-CR* non-complete responders, *SD* standard deviation, *TACE* transcatheter arterial chemoembolization

MPP was significantly higher in VT-lesions compared to RT-lesions (180.5 ± 213.6 vs 92.8 ± 21.6, *p* = 0.001). No other significant differences in baseline CT texture parameters were observed. Data is summarized in Table 2.

**Table 2:** Comparison of baseline characteristics between RT-lesions and VT-lesions

	VT-lesions	RT-lesions	*p* value
No. of HCC lesions	72	20	–
Age (range)	71.7 (49–87)	65.3 (49–88)	0.03*
Gender (%)			
Women	15 (20.8)	2 (10)	0.27
Men	57 (79.2)	18 (90)	
Cirrhosis (%)			
Yes	60 (83.3)	20 (100)	< 0.001*
No	12 (16.7)	0 (0)	
BCLC stage (%)			
A	38 (52.8)	10 (50)	0.83
B	34 (47.2)	10 (50)	
No. of HCC lesions per patient	2.7 (1–7)	1.7 (1–4)	< 0.001*
Follow-up after TACE in days (SD)	268 (± 235)	171 (± 110)	< 0.001*
I	1 (1.4)	0 (0)	
II	10 (13.9)	0 (0)	
III	2 (2.8)	0 (0)	
IVa	5 (6.9)	3 (15)	
IVb	4 (5.6)	0 (0)	
Liver segment (%)			
Left lobe	22 (30.6)	3 (15)	0.03*
V	11 (15.3)	3 (15)	
VI	8 (11.1)	0 (0)	
VII	13 (18.1)	1 (5)	
VIII	18 (25.0)	13 (65)	
Right lobe	50 (69.4)	17 (85)	
Area in mm^2^ (SD)	1062.8 (± 2308.6)	789.9 (± 1167.2)	0.61
Long axis in mm (SD)	33.9 (± 28.6)	36.9 (± 26.9)	0.68
MPP (SD)	180.5 (± 213.6)	92.8 (± 21.6)	0.001*
Entropy (SD)	6.084 (± 0.55)	5.934 (± 0.79)	0.33
Kurtosis (SD)	3.3 (± 1.1)	3.4 (± 1.0)	0.72
Skewness (SD)	− 0.1 (± 0.35)	− 0.1 (± 0.4)	0.42
Uniformity (SD)	0.0184 (± 0.008)	0.0222 (± 0.015)	0.31

Statistically significant differences are marked by an asterisk

*VT-lesions* lesions undergoing primary TACE, *RT-lesions* lesions undergoing repeated TACE, *SD* standard deviation, *TACE* transcatheter arterial chemoembolization

### Response to TACE Treatment

**Fig. 2:**
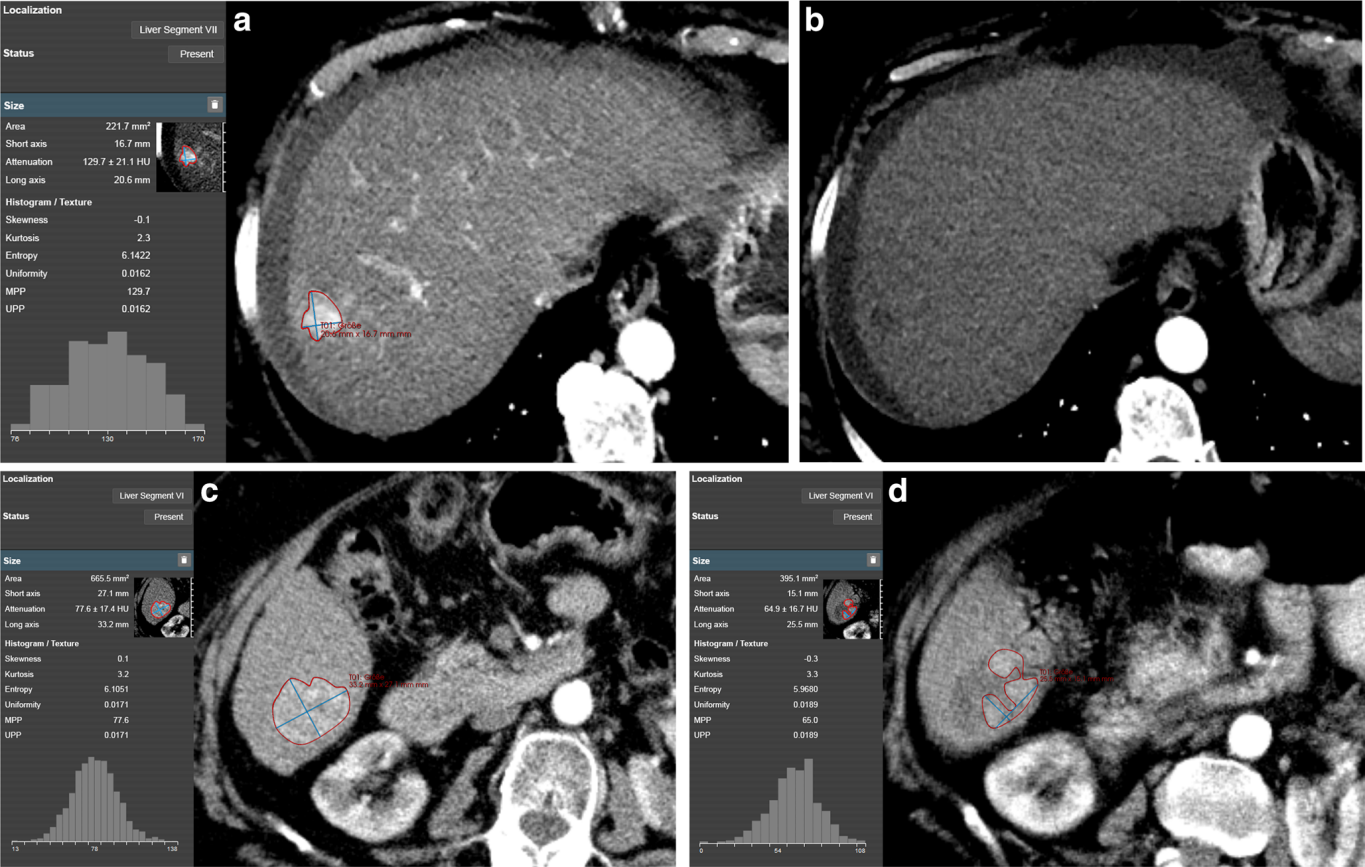
**a** Screenshot of a manually segmented HCC lesion according to mRECIST criteria on baseline arterial phase imaging in mint Lesion™ 3.0 software with automatic generation of gray-level histograms and extraction of CT texture parameters. Favorable lesion size and parameters from CT texture analysis (especially mean of positive pixels (MPP) and uniformity) render this HCC lesion likely to respond completely to transcatheter arterial chemoembolization (TACE) treatment. **b** Four weeks after TACE treatment, the HCC shows no enhancement in arterial phase (thus, no viability) resulting in complete response as per mRECIST criteria. **c** Another manually segmented HCC lesion at baseline imaging. Unfavorable lesion size, surrounding liver parenchyma (cirrhotic), and parameters from CT texture analysis (especially MPP and uniformity) at baseline imaging result in a low likelihood of complete response to TACE treatment. **d** Follow-up imaging 4 weeks after TACE treatment shows substantial residual arterial enhancement (= viable portions of the tumor), resulting in partial response as per mRECIST criteria. The patient subsequently underwent repeated TACE, and the post-treatment CT images were used as new baseline

In the primary TACE group, 38 of the 72 lesions (52.8%) showed CR on post-therapeutic imaging (example in Fig. 2); 16 lesions (22.2%) showed PD on follow-up CT. In the repeated TACE group, 8 of 20 lesions (40%) were rated as CR, while 8 lesions (40%) showed PD on follow-up imaging.

The course of each lesion over time and target response at follow-up imaging timepoints is illustrated as a swimmer plot (supplemental material). Mean time frame for overall disease follow-up was significantly longer for VT-lesions, compared to RT-lesions (268 ± 235 days vs 171 ± 110 days, *p* = 0.01). Timepoints of disease-related death or liver transplantation are visualized in the swimmer plot.

### Prediction of lesion response to primary TACE

**Fig. 3:**
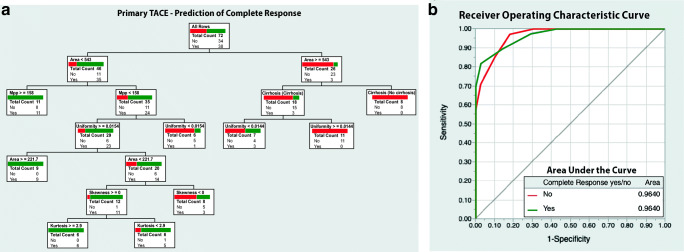
**a** Decision tree model based on texture parameters, size, and surrounding liver parenchyma (cirrhotic vs non-cirrhotic) to predict complete response prior to primary transcatheter arterial chemoembolization (TACE) treatment. **b** ROC curve with AUC values for the model

The calculated decision tree model to predict CR in VT-lesions had eight binary splits and used six parameters to accurately categorize lesions. These were as follows: area (total effect 0.744), uniformity (0.267), cirrhosis (0.145), MPP (0.019), skewness (0.003), and kurtosis (0.001). The model’s ROC curve analysis reached an area under the curve (AUC) of 0.96. Correct prediction was achieved in 64 of 72 cases, resulting in a PPV of 96.9%, an NPV of 82.5, and an accuracy of 88.9%. The model is visualized in Fig. 3.

**Fig. 4:**
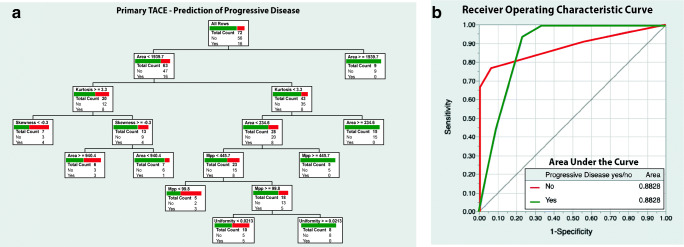
**a** Decision tree model based on texture parameters, size, and surrounding liver parenchyma (cirrhotic vs non-cirrhotic) to predict progressive disease prior to primary transcatheter arterial chemoembolization (TACE) treatment. **b** ROC curve with AUC values for the model

The decision tree model to predict PD in VT-lesions had seven binary splits and used five contributing parameters: area (0.974), kurtosis (0.201), skewness (0.078), MPP (0.004), and uniformity (0.003). ROC analysis reached an AUC of 0.88. Correct target response was predicted for 58 of 72 lesions (PPV 58.3%, NPV 85.0%, resultant accuracy 80.6%). The model is visualized in Fig. 4.

### Prediction of lesion response to Re-TACE

**Fig. 5:**
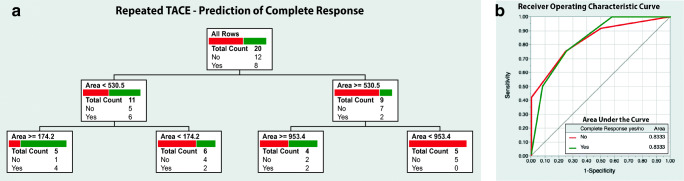
**a** Decision-tree model based on texture parameters, size, and surrounding liver parenchyma (cirrhotic vs non-cirrhotic) to predict complete response prior to repeated transcatheter arterial chemoembolization (TACE) treatment. **b** ROC curve with AUC values for the model

The decision tree model to predict CR in RT-lesions had three binary splits with only one contributing parameter (area; total effect 0.986). The model’s AUC was 0.83. Correct target response was predicted in 15 of 20 lesions (PPV 80.0, NPV 83.3%, resultant accuracy 75.0%). The model is visualized in Fig. 5.

**Fig. 6:**
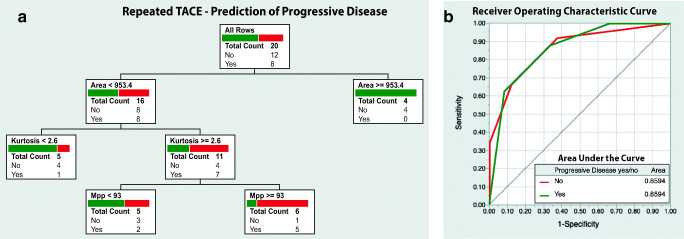
**a** Decision-tree model based on texture parameters, size, and surrounding liver parenchyma (cirrhotic vs non-cirrhotic) to predict progressive disease prior to repeated transcatheter arterial chemoembolization (TACE) treatment. **b** ROC curve with AUC values for the model

The decision tree model to predict PD in RT-lesions had three binary splits and three contributing parameters: area (0.97), MPP (0.125), and kurtosis (0.068). The AUC of the model was 0.86, and correct response was predicted for 16 of 20 lesions (PPV 83.3%, NPV 78.6%, resultant accuracy 80.0%). The model is visualized in Fig. 6. Parameter effects for each model are listed in Table 3.

**Table 3:** Total effect of lesion size and distinct CT texture analysis parameters to prediction models

Parameter	CR model 1st TACE	PD model 1st TACE	CR model Re-TACE	PD model Re-TACE
Area	0.744	0.974	0.986	0.97
MPP	0.019	0.004	0.001	0.125
Cirrhosis	0.145	0	0.001	0
Uniformity	0.267	0.003	0.001	0
Kurtosis	0.001	0.201	0.001	0.068
Skewness	0.003	0.078	0.001	0
Entropy	0	0	0.001	0

*CR* complete response, *PD* progressive disease, *MPP* mean of positive pixels, *TACE* transcatheter arterial chemoembolization

## Discussion

The aim of our study was to assess the feasibility of generating decision tree models based on pre-therapeutic CT texture parameters to predict complete response or progressive disease of HCC lesions to TACE treatment according to mRECIST criteria. Our results demonstrate that both target responses can be predicted with high accuracy for HCCs undergoing both primary TACE and repeated TACE. To our knowledge, this is the first description of nested multiparametric prediction models for this tumor entity.

Our study sample represents the two patient populations typically triaged to TACE treatment: individuals suffering intermediate stage disease for palliative loco-regional treatment and patients with early stage of disease being poor surgical candidates or undergoing bridging TACE while awaiting liver transplantation. Mean age and gender distribution of our study sample match the typical HCC epidemiology in the Western world [13].

Image-based texture analysis is an emerging methodology to gain additional quantitative information on lesion heterogeneity based on gray-level histograms. It is used to extract and process pixel distribution within a region or interest. Both CT and MRI imaging are generally suitable modalities for analyses, potentially serving as non-invasive imaging biomarkers for prognosis and treatment response [14, 15]. Many studies assessed the feasibility of this method for various tumor entities with promising results, e.g., endometrial cancer [16], pancreatic cancer [17], and non-small cell lung cancer [18]. Several published studies already focused on prediction of therapeutic response of HCCs to transcatheter arterial chemoembolization [11, 12, 19, 20]. Strong arterial enhancement, smaller tumor size, and lower homogeneity were found to be significant predictors of a complete response outcome. Our results confirm this observation, since especially the size of a lesion was the parameter with the highest total effect in all prediction models. Tumor heterogeneity, represented by the parameters’ uniformity and MPP, also proved to have relevant effects when aiming to predict target response in lesions undergoing primary TACE.

When targeting response prediction to repeated TACE, arterial enhancement seemed to be of less importance though, since it only had an effect in the model predicting progressive disease of RT-lesions, but not in the complete response model. We interpret this finding as resultant change in the underlying lesion vascularization caused by the primary TACE treatment. Due to the desired synergistic effects of vessel blockage and cytotoxic chemotherapeutic agent, the vascular bed of tumors has been damaged sufficiently to impact parenchyma perfusion. Success of repeated TACE hence does not primarily depend upon good arterial enhancement on pre-therapeutic CT imaging. This change in vascularization after primary TACE treatment is also supported by the fact that we observed significantly lower MPPs in lesions undergoing repeated TACE, compared to lesions scheduled for primary treatment. Therefore, arterial contrast enhancement per se is a less relevant prognostic parameter in HCCs undergoing repeated TACE.

A study that supports our hypothesis was performed by Fujita et al [21] in 2008, who revealed discrepancies between arterial enhancement on pretherapeutic CT scans and uptake of ethiodized oil (Lipiodol). In their study, 14.5% of tumors with poor to no enhancement on baseline CT images showed, however, moderate to complete accumulation of Lipiodol, emphasizing that success of treatment does not always correlate with lesion enhancement on baseline CT or hepatic angiography. This likely affects prediction of therapeutic effects of TACE treatment and underlines the benefit of nested multiparametric models which place impacting tissue characteristics in an additive matrix rather than single parameter–based attempts of prediction in patients suffering hepatocellular carcinoma.

Our prediction models and especially decision tree visualization with thresholds for the utilized parameters allowing binary splits are a novel paradigm to transfer CT texture analyses of HCCs into future clinical practice. Decision trees are popular in a wide range of medical and non-medical professions for a variety of reasons, easy interpretability probably being the most important advantage. In contrast to other artificial intelligence models, which also increasingly find their way into clinical practice but are usually based on non-traceable neural networks, decision tree models are comprehensible and reproducible for the user.

Besides clinical relevance, implementability into routine workflows is one of the main challenges when aiming to transfer research innovations into clinical practice. We extracted CT texture parameters from software that radiologists in our department use for longitudinal follow-up imaging in oncologic patients. As the segmentation of target lesions is performed routinely for the radiology report and CT texture parameters are computed automatically, no additional tasks would have to be performed by the interpreting radiologist. This is an advantage over analysis with additional software solutions, resulting in increased workload in daily routine. However, some of these texture analysis tools offer additional parameters, e.g., gray-level co-occurrence matrices, which may further increase prediction model accuracies.

Our study has several limitations. The study sample was rather small; however, by analyzing all treated HCCs of patients, we reached a sufficient number of lesions for modeling. CT scans were performed on two different scanner systems. Resulting possible slight differences in CT attenuation values may have caused a bias in texture parameters. Since both scanners were however manufactured by the same vendor and we consistently used the same imaging protocol, we rate this possible error of lesser importance. Segmentation of lesions was performed manually which is always prone to errors. Consensus reading of the segmented ROIs by three radiologists, two of them with long-term experience in liver imaging, should have reduced this error to a minimum. Finally, our analysis is based on 2D segmentation on axial image datasets. This is attributed to the analysis according to mRECIST criteria, even though 3D analysis of lesions would possibly have been more accurate in terms of tumor heterogeneity.

In conclusion, our study provides strong evidence that CT texture analyses of HCC lesions at baseline imaging prior to TACE may be used to accurately predict therapeutic response when using nested multiparametric decision tree models, which are easily understandable for everyone involved in the decision process of triaging patients to TACE.

## Supplementary Information

ESM 1 (PDF 899 kb)

## Abbreviations

BCLC: Barcelona Clinic Liver Cancer
CR: Complete response
HCC: Hepatocellular carcinoma
MPP: Mean of positive pixels
PD: Progressive disease
RT-lesions: Previously treated lesions undergoing repeated TACE
TACE: Transcatheter arterial chemoembolization
VT-lesions: Virgin lesions undergoing primary TACE
