# Supplementary material for: Response prediction of hepatocellular carcinoma undergoing transcatheter arterial chemoembolization: unlocking the potential of CT texture analysis through nested decision tree models
Source: Eur Radiol. 2020 Dec 3;31(6):4367–76. doi: 10.1007/s00330-020-07511-3 (PMC8128820; doi:10.1007/s00330-020-07511-3)
Supplement: Supplementary file 1 — (PDF 899 kb) [file 330_2020_7511_MOESM1_ESM.pdf]

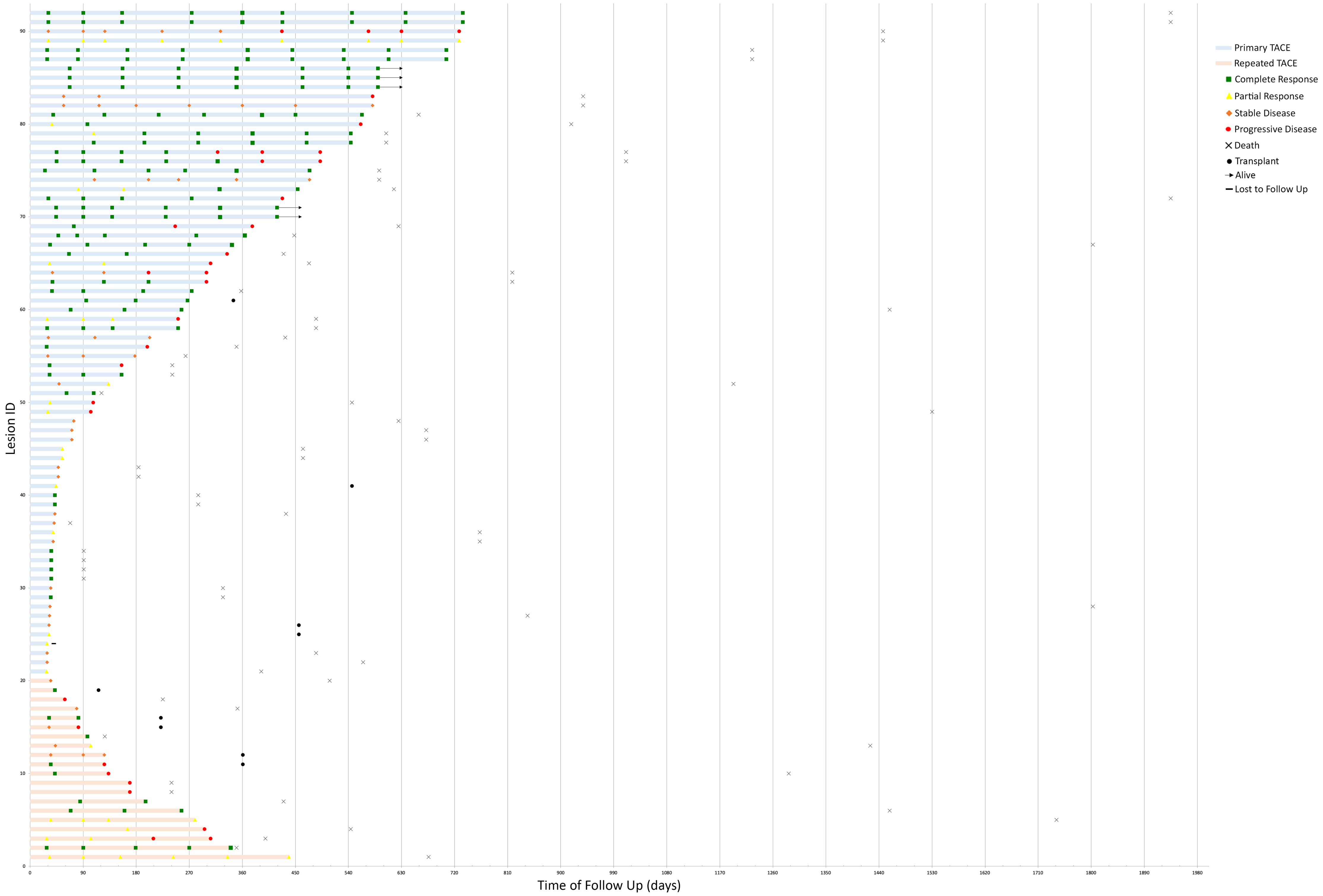
Primary TACE
Repeated TACE
Complete Response
Partial Response
Stable Disease
Progressive Disease
Death
Transplant
Alive
Lost to Follow Up
Lesion ID
0
10
20
30
40
50
60
70
80
90
Time of Follow Up (days)
0
90
180
270
360
450
540
630
720
810
900
990
1080
1170
1260
1350
1440
1530
1620
1710
1800
1890
1980

Swimmer plot depicting the course of each lesion over time (in days) and response to TACE according to mRECIST 1.1 criteria at the respective follow up timepoints. Blue bars represent course of lesions after primary TACE, orange bars depict evolution of lesions which underwent repeated TACE. TACE = Transcatheter arterial chemoembolization.
